# DDR2 Expression in Cancer-Associated Fibroblasts Promotes Ovarian Cancer Tumor Invasion and Metastasis through Periostin-ITGB1

**DOI:** 10.3390/cancers14143482

**Published:** 2022-07-18

**Authors:** Favour A. Akinjiyan, Ritu M. Dave, Emily Alpert, Gregory D. Longmore, Katherine C. Fuh

**Affiliations:** 1 Department of Obstetrics and Gynecology, School of Medicine, Washington University, St. Louis, MO 63110, USA; a.favour@wustl.edu (F.A.A.); ritudave@wustl.edu (R.M.D.); emily.r.alpert@wustl.edu (E.A.); 2 Center for Reproductive Health Sciences, Washington University, St. Louis, MO 63110, USA; 3 ICCE Institute, Washington University, St. Louis, MO 63110, USA; glongmore@wustl.edu; 4 Department of Medicine (Oncology), Washington University, St. Louis, MO 63110, USA

**Keywords:** invasion, metastasis, ovarian cancer

## Abstract

**Simple Summary:**

Ovarian cancer is the most fatal gynecological disease. Intraperitoneal metastasis contributes to complications from the disease. As such, it is important to clarify the molecular mechanisms that underlie ovarian cancer metastasis. We identified that collagen-receptor, DDR2, is an upstream regulator of periostin in cancer-associated fibroblasts and that this interaction promotes tumor metastasis.

**Abstract:**

Ovarian cancer has the highest mortality of all gynecologic malignancies. As such, there is a need to identify molecular mechanisms that underlie tumor metastasis in ovarian cancer. Increased expression of receptor tyrosine kinase, DDR2, has been associated with worse patient survival. Identifying downstream targets of DDR2 may allow specific modulation of ovarian cancer metastatic pathways. Additionally, stromal cells play a critical role in metastasis. The crosstalk between tumor and stromal cells can lead to tumor progression. We first identified that tumor cells co-cultured with DDR2-expressing fibroblasts had lower periostin expression when compared to tumor cells co-cultured with DDR2-depleted fibroblasts. We confirmed that DDR2 regulates POSTN expression in ovarian cancer-associated fibroblasts (CAFs). We found that mesothelial cell clearance and invasion by tumor cells were enhanced three-fold when DDR2 and POSTN-expressing CAFs were present compared to DDR2 and POSTN-depleted CAFs. Furthermore, DDR2-depleted and POSTN-overexpressing CAFs co-injected with ovarian tumor cells had increased tumor burden compared to mice injected with tumor cells and DDR2 and POSTN-depleted CAFs. Furthermore, we demonstrated that DDR2 regulates periostin expression through integrin B1 (ITGB1). Stromal DDR2 is highly correlated with stromal POSTN expression in ovarian cancer patient tumors. Thus, DDR2 expression in CAFs regulates the steps of ovarian cancer metastasis through periostin.

## 1. Introduction

Ovarian cancer is the deadliest gynecological malignancy. Patients with advanced disease have low five-year survival rates [1]. Ovarian cancer metastasizes through peritoneal dissemination [2] in which cancer cells attach to and clear the mesothelial cell layer in order to invade the basement membrane to form metastatic nodules [3]. In addition, the interaction between tumor cells and the surrounding stroma is crucial for tumor progression and metastasis [4,5,6]. In fact, extracellular remodeling by stromal cells, including fibroblasts, in the tumor microenvironment (TME) can contribute to metastatic signaling [7]. Deciphering molecular networks that are involved in the steps of metastasis is important for cancer therapeutic development. 

The process of tumor metastasis can be studied in vitro using 2D and 3D cultures [8]. Tumor spheroids model the process of collective migration that occurs during metastasis in vivo [9]. Tumor spheroid branching and invasion can also be used to identify pro or anti-metastatic pathways or targets [10,11,12]. In addition, the use of basement membrane gels containing collagens and laminins can further recapitulate the steps of metastasis in 3D culture [13]. 

Signaling networks involving tyrosine kinases have been shown to play a critical role in tumor progression. Discoidin Domain Receptor-2 (DDR2) is a receptor tyrosine kinase whose ligand is fibrillar collagen [14,15]. High expression of DDR2 leads to increased metastasis in various cancers [16,17,18,19,20,21,22]. Fibrillar collagen is more abundant in the stroma of ovarian cancer patients compared to ovaries of healthy controls [23]. DDR2 is overexpressed in epithelial cancer cells as they acquire an invasive phenotype [24]. Epithelial-to-Mesenchymal transition (EMT) factor, TWIST1, induces expression of DDR2 [25]. In addition, DDR2 stabilizes SNAIL and upregulates activity of matrix metalloproteinases (MMP) in ovarian cancer [19,21,25,26]. Given the role of DDR2 in cancer metastasis, studying the downstream networks that are regulated by this tyrosine kinase in tumor and stroma is important.

The metastatic potential of tumor cells is also influenced by matricellular proteins like periostin (POSTN) [27]. POSTN expression is dysregulated in inflammatory states and in malignant cells [28]. POSTN signals through the PI3K/Akt and FAK/Src pathways [29,30]. In addition, interaction between POSTN and various integrins (α_v_, β1, and β3) has been identified but direct binding has not been shown [31,32,33]. In ovarian and colorectal cancer, others have shown that overexpression of POSTN in tumor and stromal cells has been associated with poor survival, chemoresistance, tumor proliferation, and inhibition of immune cell activity [29,30,34,35,36]. However, the upstream regulatory network of POSTN in ovarian cancer is largely unknown. 

In this study, we demonstrate that DDR2 regulates POSTN expression. Using patient-derived omentum cancer-associated fibroblasts from high-grade serous ovarian cancers (Om-CAFs), we show that DDR2-depletion leads to decreased expression of POSTN. Additionally, OmCAFs with high DDR2 and POSTN expression induce tumor cell invasion and in vivo tumor implantation. As such, pathways modulated by DDR2 and POSTN can represent therapeutic targets in ovarian cancer.

## 2. Materials (or Subjects) and Methods

### 2.1. Cell Culture

COV362 (Sigma-Aldrich, Burlington, MA, USA) was maintained in DMEM Medium (Gibco, Waltham, MA, USA) supplemented with 10% heat inactivated fetal bovine serum and 1% penicillin and streptomycin. ES2 cells were obtained from NCI and maintained in McCoy’s 5A (modified) medium (Life Technologies, Carlsbad, CA, USA) supplemented with 10% heat inactivated fetal bovine serum and 1% penicillin and streptomycin. OVCAR3-TPMES cells were isolated from mouse mesentery tumor nodules in an OVCAR3TP intraperitoneal model [37]. The OVCAR3TP cells were a generous gift from Branimir I. Sikic [38]. A2780 was purchased from ATCC (Manassas, VA, USA). Cell lines were maintained at 37 °C in a 5% CO_2_ incubator. STR profiling was performed by IDEXX Bioresearch to authenticate cell lines. Mycoplasma testing was performed using MycoAlert Mycoplasma Detection Kit prior to performing experiments (Lonza, Durham, NC, USA).

### 2.2. Primary Cancer-Associated Fibroblast Cell Culture

Tumor omentum from patients with ovarian cancer was obtained at the time of tumor-debulking surgery. Cancer-associated fibroblasts were isolated as previously published [39] and cultured in DMEM with 20% FBS, 1% pen-strep, 1% MEM Non-Essential Amino Acids (Gibco, Waltham, MA, USA), and 2% MEM Vitamins (Gibco, Waltham, MA, USA). After 7–14 days, attached and proliferating cells were passaged and used for experiments. CAF 43, 68, 94, and 106 were all obtained from patients with advanced stage, high-grade serous ovarian or fallopian tube cancer. All the patients who participated in this study provided written informed consent for the collection and research use of their materials, and the use of these samples was approved by the Washington University Institutional Review Board (IRB 201309050). 

### 2.3. Secretome Protein Analysis

Patient-derived normal omental fibroblasts expressing DDR2 (NOF siCTRL) and fibroblasts with DDR2 knockdown (NOF siDDR2) were used. 24 h conditioned media from NOF siCTRL and NOF siDDR2 cells was collected for analysis. In addition, 24 h conditioned media from NOF siCTRL and NOF siDDR2 cells was used as chemoattractant in an invasion assay with serum starved ES2 cells plated on Matrigel in the Boyden chamber. After 24 h invasion assay, NOF siCTRL + ES2 media and NOF siDDR2 + ES2 media were also collected for mass spectrometry analysis by MS Bioworks (Ann Arbor, MI, USA). Fold change in protein expression was calculated as the ratio of protein expression (normalized spectral abundance factor) in control condition to protein expression in DDR2-depleted condition. *p*-value < 0.05 and Fold change 2.0 or 0.5 were used as cutoff values.

### 2.4. Genetic Knockdown and Overexpression

The oligos for human DDR2 shRNA, 5′-GCCAGATTTGTCCGGTTCATT-3′ and 5′-GCCAAGTGATTCTAGCATGTT-3′, and control, 5′-CCTAAGGTTAAGTCGCCCTCGCTC-3′, were cloned into the pLKO vector and infected cells were selected in puromycin (Sigma, St. Louis, MO, USA). The following siRNAs were used: siControl-ON-TARGETplus Non-targeting pool (Dharmacon, Lafayette, CO, USA), siDDR2-human ON-TARGETplus DDR2 siRNA SMARTpool (Dharmacon, Lafayette, CO, USA) and siPOSTN-ON-TARGETplus Human POSTN siRNA SMARTpool (Dharmacon, Lafayette, CO, USA). To overexpress POSTN, we used Periostin (POSTN) (NM_001135935) Human Tagged ORF Clone (Origene Technologies, Rockville, MD, USA) and pCMV6-Entry Empty Mammalian Expression Vector (Origene Technologies, Rockville, MD, USA). For all genetic knockdown and overexpression experiments, polyclonal populations were tested for decreased DDR2 expression levels by immunoblot analysis.

### 2.5. Tumor Cell Spreading 

CAFs were embedded in 1 mg/mL Matrigel (Corning, Corning, NY, USA) with 30 μg/mL collagen or 0.5 mg/mL polymerized collagen and incubated at 37 °C, 5% CO_2_ for 24–48 h. For all experiments, we polymerized collagen at pH 7 and temperature 37 °C. Tumor spheroids were formed with 300 ES2 cells/well and 10 μg/mL fibronectin (Corning) and incubated in ultra-low attachment plates (Corning, Corning, NY, USA) at 37 °C, 5% CO_2_ overnight. On the day of the experiment, the tumor spheroids were transferred onto the CAF-embedded gel. Time zero, 24 h, and 48 h tumor spreading pictures were acquired and sprout area was measured using the WIMASIS WimSprout analysis platform. Sprout area ratio was calculated by the final sprout area divided by initial sprout area. 

### 2.6. 3D Collagen Invasion

Similar to previously published literature [26,40], 50,000 ES2 tumor cells were embedded in a collagen plug (1 mg/mL concentration) and incubated on a 24-well plate coated with 3D collagen type 1. Upon polymerization of the cell-containing collagen plug, another layer of collagen was added to encapsulate the cell plug. Images were taken on a brightfield microscope with 4× objective at time zero, and every 24 h for a total of 7 days. Invasion area was measured as the total area of cell invasion from the original boundary using ImageJ. Conditioned media from CAFs was used to form all 3D collagen, including the cell plug. Conditioned media was also added atop the gels after polymerization of the topmost collagen layer. 

### 2.7. Mesothelial Cell Clearance

Human primary mesothelial cells (HPMCs) were cultured in 6-well plates (Techno Plastic Products, Trasadingen, Switzerland) until >80% confluence was reached. HPMC and CAFs (1:1 ratio) were labeled with CMFDA-green (Molecular Probes, Eugene, OR, USA), washed with PBS, and incubated with fresh cell culture media. Spheroids were prepared as described above but ES2 cells were labelled with CMTPX-red (Molecular Probes) prior to plating in the ultra-low attachment plates. Plates containing labelled cells were incubated at 37 °C, 5% CO_2_ overnight. Spheroids were added onto the HPMC and CAF monolayer at the microscope and time lapse images were acquired from time zero until 20 h. Clearance ratio was determined by measuring the total area cleared by the spheroid in ImageJ, divided by the size of the spheroid at time zero. 

### 2.8. Attachment Assay

HPMCs and CAFs (1:1 ratio) were plated in a 96 well plate, 5 × 10^5^ cells/well total and incubated overnight at 37 °C, 5% CO_2_. ES2 cells were stained with CMFDA-green. HPMC and CAFs were washed with PBS, and 100 µL of the labeled ES2 cell suspension (5 × 10^4^ cells) were added to each well and incubated at 37°C for 1 h. After the incubation, the total fluorescence at Ex/Em = 494/517 nm was measured using an Infinite M200Pro plate reader (Tecan, Männedorf, Switzerland). Wells were washed with PBS twice to remove non-adherent cancer cells. PBS was added to the wells and fluorescence was measured again. Percent attachment was determined by dividing the corrected (background subtracted) fluorescence of adherent cells by the total corrected fluorescence of cells added to each microplate well and multiplying by 100.

### 2.9. Transwell Invasion Assay

1 mg/mL Matrigel (Corning) was plated in Boyden chambers (Corning) and allowed to polymerize. 24 h conditioned media from CAFs was used as a chemoattractant in invasion assay. 10,000 ES2 cells were plated in 100 µL media atop the polymerized gel. ES2 cells were allowed to invade for 48 h. Matrigel was removed from the chambers using a cotton swab. The membrane was fixed, stained, and imaged. Cells were quantified by counting number of invaded cells per high powered field at 20×. 

### 2.10. Wound Healing Assay

ES2 cells were plated at 5 × 10^5^ cells/well in a 12-well plate (Techno Plastic Products) and incubated at 37 °C, 5% CO_2_ overnight. A vertical scratch was made on the ES2 monolayer cells using a toothpick. Cells were rinsed with PBS to remove dislodged cells and 24 h conditioned media from CAFs was added to the ES2 cells. The plate was then incubated at 37 °C, 5% CO_2_ for 24 h. Time zero and 24 h pictures were taken using a microscope at 4×. Migration area was determined using the wound healing size tool plugin in ImageJ. Percent migration was calculated as the percent change in wound healing area over 24 h. 

### 2.11. Statistical Analysis

Graphpad Prism 9 was used for statistical analysis. All statistical tests were performed using the Student’s t-test with *p*-values of <0.05 considered statistically significant. 

### 2.12. Western Blot Analysis

Protein lysates, collected in 9 mol/L urea and 0.075 mol/L Tris, pH 7.6, were quantified using a Bradford protein quantification assay. Samples were normalized for protein concentration. Protein visualization was performed using a Jess Automated Protein Analysis system (BioTechne, Minneapolis, MN, USA). Antibody dilutions were as follows: DDR2 (1:50, Cell Signaling Technology, Danvers, MA, USA), POSTN (1:200 Adipo-gen, San Diego, CA, USA), pAKT (1:50, Cell Signaling Technology, Danvers, MA, USA), pSrc (1:50, Cell Signaling Technology, Danvers, MA, USA), ITGB1 (1:50, Thermofisher, Waltham, MA, USA), Actin (1:200, Sigma-Aldrich, Burlington, MA, USA). For signaling pathway analysis, CAFs were embedded in 1 mg/mL polymerized collagen 1 overnight prior to collecting protein lysates. 

### 2.13. Co-Immunoprecipitation Assay

CAFs were transfected with Flag-tagged constructs (Origene Technologies, Rockville, MD, USA) using TransIT-LT1 reagent (Mirus Bio, Madison, WI, USA). Cells were collected 36 h after transfection, lysed, and protein quantification was performed using Bradford. Protein concentration was normalized between samples and equilibrated FLAG beads (Sigma Aldrich, Burlington, MA, USA) was added for overnight incubation at 4 °C. Pulldown assay was performed according to manufacturer’s protocol (Sigma Aldrich, Burlington, MA, USA). Interacting proteins were eluted using SDS-PAGE sample buffer, and immunoblot for ITGB1 was performed.

### 2.14. cDNA Preparation and Quantitative Real-Time PCR

To isolate total cellular RNA, the RNAEasy plus mini kit (Qiagen, Germantown, MD, USA) was used. 1 μg of the RNA was used to make cDNA. Real-time PCR reactions were done using the SYBR Green PCR Master Mix (Applied Biosystems, Waltham, MA, USA) in the ABI detection system (Applied Biosystems). The experiments were carried out in triplicate for each data point. Gene expression was quantified using the 2−ΔΔCt method. RT-PCR primers used were as follows: DDR2 (Fwd 5′-TCA CCC AGA CCC ATG AAT AC-3′, Rev 5′-GGG AAG GAA ATG GCA TTA GG-3′), POSTN (Fwd 5′-GAT GGA GTG CCT GTG GAA ATA-3′, Rev 5′-GTT TCT CCA CCT CCA GTA GAA AT-3′).

### 2.15. Xenograft Model of Ovarian Cancer

All procedures involving animals and their care were performed in accordance with the guidelines of the American Association for Accreditation for Laboratory Animal Care and the U.S. Public Health Service Policy on Human Care and Use of Laboratory Animals. All animal studies were also approved and supervised by the Washington University Institutional Animal Care and Use Committee in accordance with the Animal Welfare Act, the Guide for the Care and Use of Laboratory Animals and NIH guidelines (Protocol 20-0378). For genetic studies, CAF68hT shSCRM + ES2, CAF68hT shDDR2 + ES2, CAF68hT shDDR2 POSTN OE + ES2, CAF68hT shDDR2 Empty Vector + ES2, ES2 alone or CAF68hT shSCRM alone were injected intraperitoneally (i.p.) at ratio 1:1 CAF:tumor amount with a total of 5 × 10^6^ cells in 0.2 mL of PBS into female 6- to 8-week old (n = 4–5 per group) NCr nude mice (Taconic). Mice were monitored for adverse events and sacrificed with CO_2_ exposure and cervical dislocation at 15 days after i.p. injection. Necropsy with tumor burden assessment was performed. At the completion of the experiment, aggregate tumor weight was recorded for each group.

### 2.16. Immunohistochemistry and Tumor Microarray

Ovarian human tissue microarrays were obtained from US Biomax (208) (Rockville, MD, USA) which contained normal, primary, and metastatic tumors. Slides were deparaffinized with xylene, then rehydrated and unmasked following standard immunohistochemical methods. Antibodies used were DDR2 1:500 (R&D systems, Minneapolis, MN USA) and POSTN 1:200 (Abcam, Cambridge MA, USA). Antigen–antibody complexes were visualized using the VECTASTAIN ABC system (Vector Laboratories, Newark, CA, USA) and DAB Substrate Kit for Peroxidase (Vector Laboratories, Newark, CA, USA) following the manufacturer’s protocol. Slides were counterstained in hematoxylin. DDR2 and POSTN staining in the stroma was scored microscopically according to the percentage of cells positive for DDR2 or POSTN expression both by intensity and percentage of cells with expression (0 for absent, 1–1.99 for 1%–40%, 2–2.99 for 40–60%, and 3–3.99 for >60%). For each protein, two cores were stained for each patient and the average score was used for the correlation studies. There were two blinded, independent scores obtained for DDR2 and POSTN. Correlation was performed in Graphpad prism and Spearman rho coefficient and *p*-value were reported.

### 2.17. TCGA RNA Co-Expression 

Using cbioportal.org, we accessed The Cancer Genome Atlas. We used TCGA Firehose Legacy cohort for ovarian cancer for our analyses. A total of 606 ovarian cancer samples were included in this study. Gene co-expression was analyzed for POSTN. Spearman rho coefficient and *p*-value were reported.

## 3. Results

### 3.1. CAFs Promotes Ovarian Tumor Spheroid Branching through DDR2 

During metastasis, tumor cells must undergo attachment, clearance, and invasion of the basement membrane [41,42]. A critical component that spans multiple steps of metastasis is the ability for ovarian tumor spheroids to branch in order to migrate. To determine whether CAFs would enhance tumor spheroid branching, we placed tumor spheroids above CAFs mixed in basement membrane gels or above basement membrane gels without CAFs. We monitored spheroid branching over 24 h and found that the ovarian tumor spheroids branch more in CAF-containing basement membrane gels compared to gels without CAFs (Supplementary Materials Figure S1A–C). 

**Figure 1:**
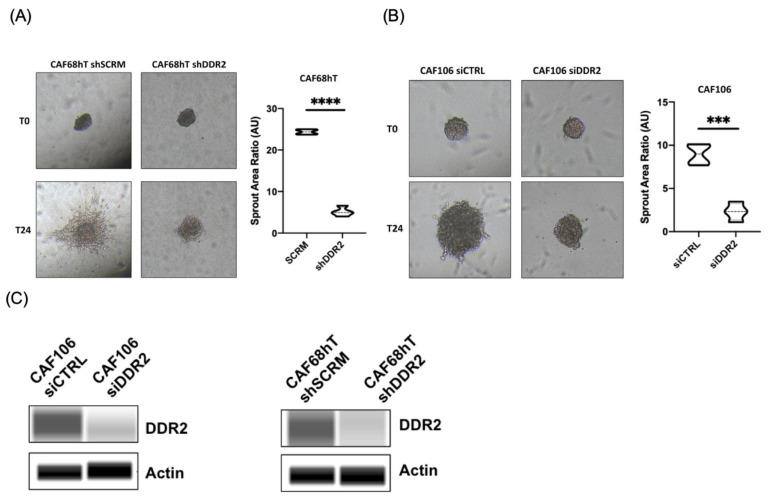
DDR2 depletion in CAFs leads to decreased tumor spheroid spreading using (**A**) CAF68hT in polymerized collagen 1 and (**B**) CAF106 in Matrigel. (**C**) Western blot showing DDR2 knockdown. *** *p* < 0.001, **** *p* < 0.0001.

We had previously identified that tumor DDR2 regulates metastasis in ovarian cancer [25]. We next asked whether the action of DDR2 in OmCAFs can contribute to tumor metastasis [17]. Since fibrillar collagen, such as collagen 1, is a ligand for DDR2 [14], we placed DDR2-expressing ES2 spheroids on CAF-containing collagen type 1 gels. Spheroids cultured with DDR2-expressing CAFs had increased sprout area compared to spheroids cultured with DDR2-depleted CAFs (Figure 1A). Increased sprout area was also observed in spheroids cultured with DDR2-expressing CAFs in Matrigel which contains collagen IV and laminin (Figure 1B, Supplementary Materials Figure S1D). DDR2 knockdown in CAFs was confirmed by immunoblotting (Figure 1C).

### 3.2. Fibroblast DDR2 Expression Regulates Periostin (POSTN) Levels

Given we identified that DDR2 expression in CAFs can further promote metastasis through regulation of tumor spheroid branching, we asked whether there were particular secreted proteins that contributed to fibroblast regulation of metastasis. Specifically, we were interested in the proteins secreted by tissue-resident fibroblasts that were induced by exposure to tumor cells. Upon exposure to tumor cells, these tissue-resident fibroblasts can transition to CAFs in vivo [43]. Thus, we collected media from tissue-resident fibroblasts cultured from normal omentum that we will refer to as normal omental fibroblasts (NOFs). We characterized our NOF and CAFs using immunoblotting for established fibroblast markers, fibroblast activated protein, and FSP/S100a4 (Supplementary Materials Figure S2A). These NOFs were co-cultured with ovarian cancer tumor cells using the same conditions for an invasion assay. Specifically, ES2 cells were placed on top of Matrigel with conditioned media from NOF siCTRL or NOF siDDR2 as the chemoattractant. After 24 h of invasion, there was less tumor cell invasion when conditioned media from NOFsiDDR2 was used as the chemoattractant. The post-invasion chemoattractant media (NOF siCTRL + ES2 and NOF siDDR2 + ES2), as well as conditioned media from NOFs alone, was sent for mass spectrometry analysis for differential expression of secreted factors. 

**Figure 2:**
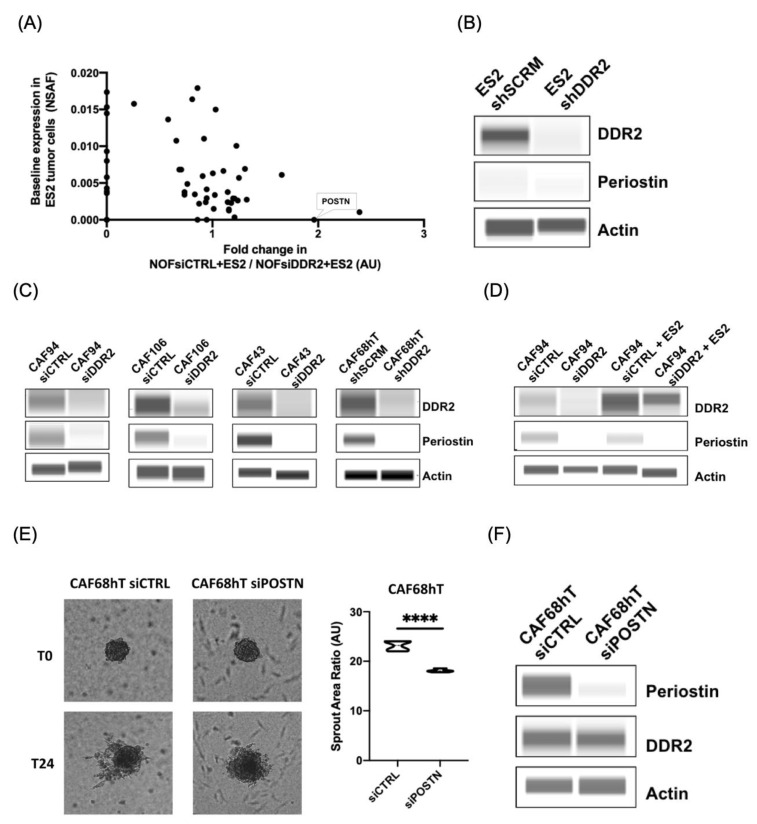
Periostin is decreased in DDR2-depleted fibroblasts co-cultured with tumor cells. (**A**) Mass spectrometry secretome analysis of DDR2-expressing and DDR2-depleted fibroblasts exposed to tumor cells. (**B**) Western blot showing that periostin is not expressed in ES2 tumor cells. (**C**) Western blot validation showing decreased periostin levels in DDR2-depleted CAFs. (**D**) DDR2-dependent decrease in periostin levels is more pronounced when CAFs are co-cultured with tumor cells. (**E**) Decreased tumor spheroid spreading observed upon depletion of POSTN in CAFs and (**F**) Western blot showing POSTN knockdown. **** *p* < 0.0001.

From the mass spectrometry analysis, 45 secreted proteins were identified. Of particular interest was periostin (POSTN), which was found to have a significantly higher expression in DDR2-expressing NOFs co-cultured with ES2 tumor cells (NOF siCTRL + ES2) compared to DDR2-depleted NOFs co-cultured with tumor cells (NOF siDDR2 + ES2) (Fold change = 2.0, *p* = 0.03) (Figure 2A). Of note, from this analysis, POSTN was not secreted at baseline in ES2 tumor cells as seen in Figure 2A. These findings are compared to DDR2-expressing NOFs that had a small decrease in POSTN secretion compared to DDR2-depleted NOFs (Fold change of 0.68, *p* = 0.03, data not shown). This was below our fold change cutoff and thus not considered significant.

We identified that ES2 tumor cells do not express POSTN (Figure 2B) and this is consistent with the lack of secreted POSTN (Figure 2A) from ES2 cells. We identified that POSTN had significantly higher expression in the DDR2-expressing NOFs co-cultured with ES2 tumor cells compared to conditioned media of NOF alone or DDR2-depleted NOFs co-cultured with ES2 tumor cells. Next, we asked whether there was a functional significance for POSTN in DDR2-expressing CAFs. To determine if DDR2 expression in CAFs regulates POSTN levels, we examined POSTN levels in DDR2-depleted CAFs by immunoblotting and quantitative PCR. 

Using a collection of patient-derived CAFs and multiple short hairpin RNA (shRNA) targeting DDR2, we showed that DDR2-depleted CAFs had lower POSTN expression levels (Figure 2C, Supplementary Materials Figure S2B). DDR2-depleted CAFs co-cultured with ES2 tumor cells had further reduction in POSTN levels compared to DDR2-expressing CAFs co-cultured with ES2 cells (Figure 2D, Supplementary Materials Figure S2C). This effect is likely CAF-specific given that ES2 cells do not express POSTN (Figure 2B). We aimed to identify if DDR2 expression in tumor cells regulates POSTN expression, so we performed immunoblotting on a panel of DDR2-expressing and DDR2-depleted cells. DDR2 expression in tumor cells did not correlate with POSTN expression (Supplementary Materials Figure S2D). These data show that DDR2 regulates POSTN expression in CAFs but that there is no correlation between DDR2 and POSTN expression in tumor cells.

We used the tumor spheroid assays to determine whether POSTN expression in CAFs influences tumor spheroid branching. We cultured ES2 spheroids on POSTN-expressing CAF-containing gels or POSTN-deficient CAF-containing gels. Spheroids cultured on POSTN-expressing CAF-containing gels had increased sprout area compared to spheroids cultured with POSTN-depleted CAF-containing gels (Figure 2E). POSTN knockdown was confirmed by immunoblotting (Figure 2F). This result indicates that tumor spheroid branching is decreased on POSTN-deficient CAF-containing gels. 

### 3.3. Regulation of Periostin by DDR2 Promotes the Steps of Tumor Metastasis in Cell-Based Assays 

**Figure 3:**
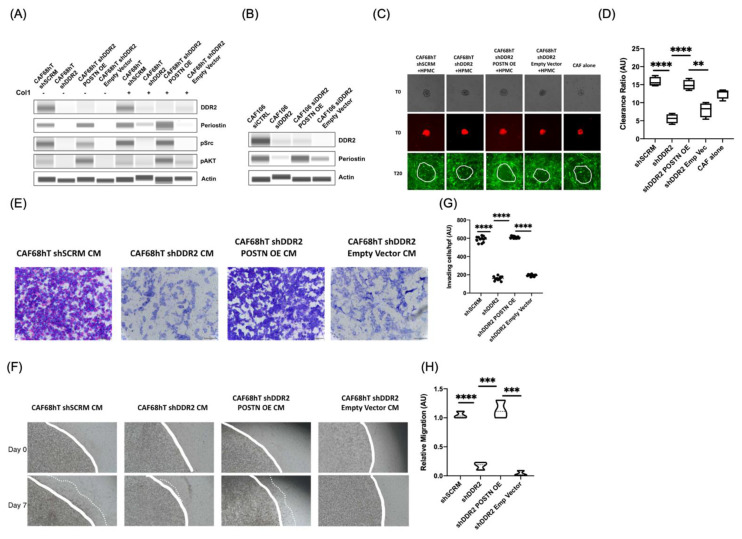
DDR2 and POSTN promote tumor metastasis in cell-based assays. (**A**) Western blot showing increase in pSrc and pAKT in POSTN overexpression in DDR2-depleted CAFs. (**B**) Western blot showing POSTN overexpression in DDR2-depleted CAF106. (**C**) Mesothelial and CAF clearance by tumor cells using DDR2 knockdown and POSTN overexpression CAFs. (**D**) Analysis of assay in (**C**). (**E**) Matrigel transwell tumor invasion assay with ES2 cells using conditioned media from DDR2 knockdown and POSTN overexpression CAFs. (**F**) 3D Collagen tumor cell migration assay using conditioned media from DDR2 knockdown and POSTN overexpression CAFs. (**G**) Analysis of assay in (**E**,**H**) Analysis of assay in (**F**). ** *p* < 0.01, *** *p* < 0.001, **** *p* < 0.0001.

Tumor attachment, clearance, and invasion through the basement membrane is necessary for metastasis [41,42]. To determine if DDR2′s regulation of periostin in CAFs influences tumor metastasis in cell-based assays, we performed attachment, clearance, and invasion assays. In order to test the effect of DDR2 and POSTN on tumor metastasis, we used DDR2-expressing (CAF shSCRM) and DDR2-depleted CAFs (CAF shDDR2) as well as DDR2-depleted, periostin-overexpressing (CAF shDDR2 POSTN OE), and transfection control (CAF shDDR2 Empty Vector) CAFs. (Figure 3A,B). 

Tumor cell attachment is the initial step of peritoneal metastasis in ovarian cancer [3]. We aimed to study the effect of DDR2′s modulation of POSTN in CAFs on tumor cell attachment. For the attachment assay, we cultured human primary mesothelial cells (HPMC) with DDR2-expressing (CAF shSCRM), DDR2-depleted (CAF shDDR2), DDR2-depleted + periostin-overexpressing (CAF shDDR2 POSTN OE), or DDR2-depleted + empty vector (CAF shDDR2 Empty Vector) CAFs. Fluorescent-labeled ES2 cells were allowed to attach for 1 h after which unattached cells were removed. We observed a decrease in tumor cell attachment in DDR2-depleted CAF condition compared to DDR2-expressing CAF condition, however these differences were not statistically significant (Supplementary Materials Figure S3A). This data suggests that DDR2 and POSTN expression in CAFs may not play a critical role in tumor cell attachment. 

Upon attachment to the mesothelial cells, tumor cells must clear through mesothelial cells in order to invade into the underlying stroma [3,6]. We aimed to define the role of DDR2′s modulation of POSTN in CAFs on tumor cell clearance of HPMCs. For the clearance assay, we cultured CAFs with HPMCs, added ES2 spheroids and monitored clearance by tumor cells for 20 h. We observed decreased clearance for spheroids cultured with DDR2-depleted CAFs compared to those with DDR2-expressing CAFs (Figure 3C). POSTN overexpression in DDR2-depleted CAFs led to an increase in clearance ratio compared to DDR2-depleted CAFs alone (Figure 3D). This data suggests that DDR2 and POSTN expression in CAFs promote tumor cell clearance of mesothelial cells.

After clearance of the mesothelial cells, tumor cells can invade into the underlying basement membrane and migrate within the collagen-rich extracellular matrix. We aimed to define the role of DDR2′s modulation of POSTN in CAFs on tumor cell invasion into the basement membrane and ECM migration. For the Matrigel transwell invasion assay, we used conditioned media from DDR2-expressing, DDR2-depleted, and DDR2-depleted + POSTN-overexpressing CAFs as chemoattractant for tumor cell invasion. ES2 cells were allowed to invade through a Matrigel plug. Since collagen 1 is a ligand of DDR2, we also studied the effect of DDR2′s modulation of POSTN on tumor cell migration through a 3D collagen extracellular matrix (ECM). In both the Matrigel transwell invasion and 3D collagen migration assays, we observed decreased tumor cell invasion in the DDR2-depleted CAF condition compared to DDR2-expressing CAFs (Figure 3E,F). In addition, overexpressing POSTN in DDR2-depleted CAFs led to an increase in tumor cell invasion (Figure 3G,H). These data indicate that DDR2 signaling through POSTN is important for tumor cell invasion through the basement membrane as well as migration through the ECM.

### 3.4. Regulation of Periostin by DDR2 Increases Tumor Spreading and Proliferation

**Figure 4:**
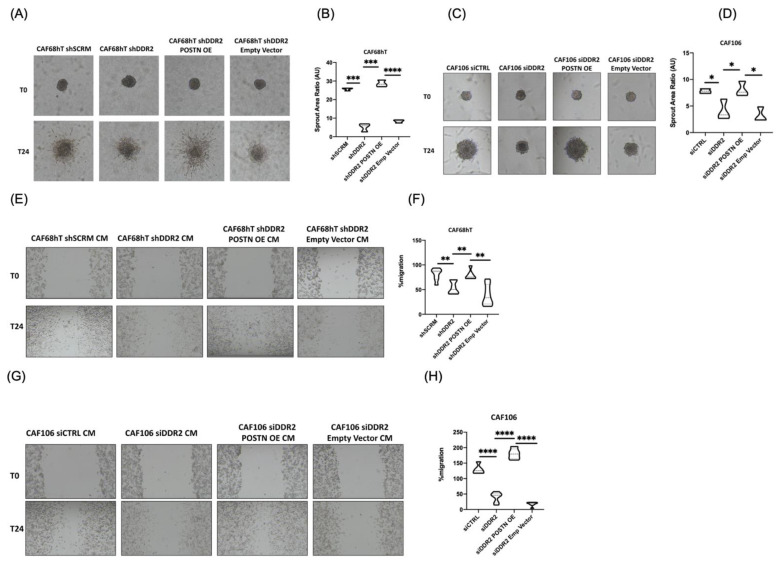
DDR2 and POSTN promote tumor migration and spreading. (**A**) Increased tumor spheroid spreading with POSTN overexpression in DDR2-depleted CAF68hT in collagen 1 gel. (**B**) Analysis of assay in (**A**). (**C**) Increased tumor spheroid spreading with POSTN overexpression in DDR2-depleted CAF106 in Matrigel. (**D**) Analysis of assay in (**C**). (**E**) Increased tumor migration and wound healing with POSTN overexpression in DDR2-depleted CAF68hT. (**F**) Analysis of assay in (**E**). (**G**) Increased tumor migration and wound healing with POSTN overexpression in DDR2-depleted CAF106, and (**H**) Analysis of assay in (**G**). * *p* < 0.05, ** *p* < 0.01, *** *p* < 0.001, **** *p* < 0.0001.

The ability of a tumor to proliferate and spread is necessary for its metastatic potential. In order to determine the role of DDR2′s modulation of POSTN on tumor cell spreading and proliferation, we performed spheroid spreading and wound healing assays. The tumor spreading assay was performed on polymerized collagen 1. Spheroids cultured with DDR2-depleted CAFs had decreased sprout area ratio, as observed before (Figure 1A and Figure 4A). However, overexpression of POSTN in DDR2-depleted CAFs led to an increase in tumor spreading as measured by sprout area ratio (Figure 4A,B). Similar findings were observed when Matrigel was used for the tumor spreading assay (Figure 4C,D, Supplementary Materials Figure S4A,B).

Tumor cell invasion through the basement membrane is in part influenced by its ability to migrate. To determine the effect of DDR2′s modulation of POSTN on tumor cell migration and proliferation, we performed wound healing assays with ES2 tumor cells and CAF conditioned media. A small scratch was introduced in ES2 cells cultured in a monolayer. Dislodged cells were removed and CAF conditioned media was added to the ES2 cells. Conditioned media from DDR2-depleted CAFs showed decreased ability to induce ES2 tumor cell migration and proliferation (Figure 4E,F). Overexpression of POSTN in DDR2-depleted CAFs led to an increased ability to promote tumor cell migration and proliferation (Figure 4E–H). These data indicate that DDR2 function through POSTN is important for tumor migration and proliferation. 

### 3.5. Regulation of POSTN by DDR2 Influences PI3K/Akt and Src Pathways

POSTN has been shown to signal through the PI3K/Akt and Src pathways in various cancers [29,35]. PI3K/Akt and Src pathways are also downstream of DDR2 signaling [26]. To determine if DDR2′s regulation of POSTN modulates the PI3K/Akt and Src pathways, we depleted DDR2 in patient-derived CAFs. We also overexpressed POSTN in DDR2-depleted CAFs to test the effects POSTN expression in a DDR2-dependent manner. We cultured these CAFs on plastic or polymerized collagen 1 and performed immunoblotting for pAKT and pSrc. DDR2 depleted CAFs had lower pAKT and pSrc levels (Figure 3A). However, overexpression of POSTN in DDR2-depleted CAFs increased pAKT and pSrc levels back to baseline (Figure 3A). This data indicates that DDR2 and POSTN acts upstream of the PI3K/Akt and Src pathways.

### 3.6. DDR2 Regulates Integrin β1 (ITGB1) Which Binds Directly to Periostin 

**Figure 5:**
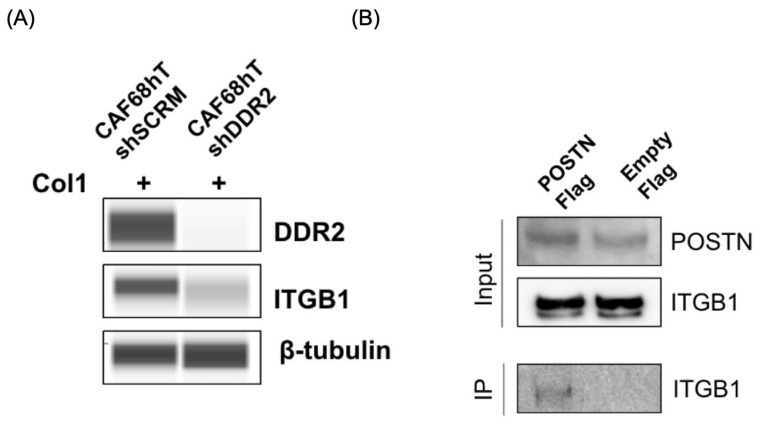
DDR2 regulates ITGB1, which is a direct binding partner of POSTN. (**A**) Immunoblot showing decreased ITGB1 expression in DDR2-depleted CAFs, (**B**) Co-immunoprecipitation of ITGB1 using FLAG-tagged POSTN and empty control constructs.

We have previously shown that the action of DDR2 in breast cancer CAFs is important for ITGB1 activity [44]. Prior work also suggests that periostin interacts with various integrin family proteins (α_v_, β1, and β3) [31,32,33]. To determine if DDR2 regulates ITGB1 expression in OmCAFs, we performed immunoblot on collagen-stimulated CAFs with or without DDR2 expression. DDR2-depleted CAFs had decreased expression of ITGB1 compared to DDR2-expressing CAFs (Figure 5A). We also aimed to determine if POSTN binds directly to ITGB1 so we performed a co-immunoprecipitation assay using FLAG-tagged POSTN and control vectors. We identified that POSTN binds directly to ITGB1 (Figure 5B). The regulatory network that connects DDR2 and POSTN includes collagen-binding receptor, ITGB1 (Supplementary Materials Figure S5). These data suggest that DDR2 controls ITGB1 expression and POSTN’s binding to ITGB1 may reflect the mechanism by which DDR2 regulates POSTN. 

### 3.7. Regulation of Periostin by DDR2 Promotes Tumor Implantation In Vivo 

**Figure 6:**
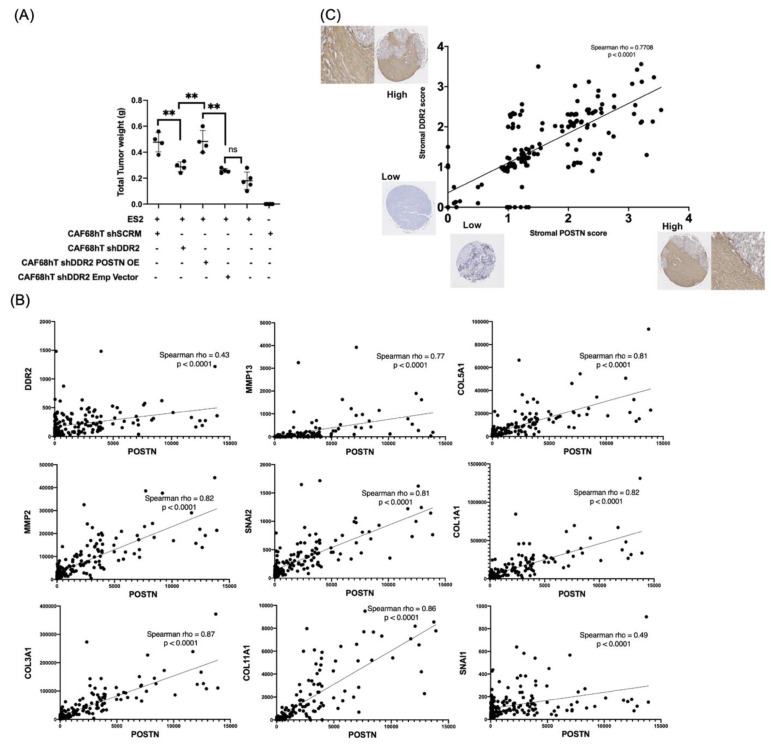
DDR2 and POSTN promote in vivo tumor implantation. (**A**) Xenograft model showing increased tumor burden in mice co-injected with DDR2-depleted, POSTN-overexpressing CAFs and ES2 tumor cells. (**B**) Correlation between POSTN mRNA expression and DDR2 (and known ligands/targets) mRNA expression using TCGA data, and (**C**) Tumor microarray IHC data showing correlation between POSTN protein expression and DDR2 protein expression. ** *p* < 0.01.

To determine if DDR2′s regulation of periostin in CAFs has an effect on tumor cell implantation in vivo, we injected ES2 cells and patient-derived CAFs into NCr nude mice and monitored ability to implant and form metastatic nodules in an intraperitoneal xenograft model. We sacrificed the mice after 15 days and compared tumor weight within the groups. Mice injected with DDR2-depleted CAFs (CAF shDDR2 + ES2) had decreased tumor burden compared to mice injected with DDR2-expressing CAFs (CAF shSCRM + ES2) (Figure 6A). Overexpression of POSTN in DDR2-depleted CAFs (CAF shDDR2 POSTN OE + ES2) led to increased tumor burden in our xenograft model. This data suggests that regulation of POSTN by DDR2 in CAFs is important for tumor metastasis in vivo. 

### 3.8. POSTN Expression Correlates with DDR2 Expression in Patient Tumors

We aimed to determine if there was a correlation between DDR2 and POSTN mRNA expression. Using The Cancer Genome Atlas–Firehouse Legacy study, we analyzed 606 ovarian cancer samples. We determined correlation between POSTN expression and DDR2 as well as known ligands and targets of DDR2, including collagen isoforms, MMPs, and SNAIL [26,45]. POSTN expression was strongly correlated with COL1A1 (Spearman rho = 0.82, *p* < 0.0001), DDR2 (Spearman rho = 0.43, *p* < 0.0001), and MMP2 (Spearman rho = 0.82, *p* < 0.0001) expression, among others (Figure 6B). 

To determine if DDR2 protein expression was correlated with POSTN protein expression in ovarian cancer patients, we performed immunohistochemistry on a tumor microarray with specimens from 180 patients with primary and metastatic tumor sites. Cores were stained for DDR2 and POSTN and scored blindly for stromal expression. Stromal DDR2 protein expression was highly correlated with stromal POSTN protein expression in ovarian cancer patients (Spearman rho = 0.77, *p* < 0.0001) (Figure 6C, Supplementary Materials Figure S5A,B). These data suggest that tumors from ovarian cancer patients with high stromal DDR2 expression also have high stromal POSTN expression. 

## 4. Discussion 

To the best of our knowledge, this is the first study to show that DDR2 regulates POSTN. We identify that the interaction between DDR2 and POSTN is important in CAFs and promotes tumor metastasis in ovarian cancer. Additionally, we confirm that DDR2 and POSTN signal through the PI3K/AKT and Src pathways. Furthermore, we show that POSTN binds directly to ITGB1 and that ITGB1 expression in OmCAFs is regulated by DDR2. DDR2 and periostin signaling in CAFs promotes tumor migration, clearance, proliferation, and invasion. POSTN and DDR2 protein expression, as well as that of known ligands and targets of DDR2, are highly correlated. 

Ovarian cancer metastasizes throughout the peritoneal cavity. Patients with high grade serous ovarian cancer (HGSOC) have a worse prognosis compared to other histologies [46]. HGSOC is genetically characterized by the presence of a TP53 mutation [47]. We derived primary, high-grade serous ovarian cancer-associated fibroblasts (HGSOC-CAFs) in this study from the omentum of ovarian cancer patients [39]. To determine whether DDR2-expressing CAFs regulated tumor cell invasion, we utilized the DDR2-expressing ES2 tumor cell line. The ES2 cell line is histologically characterized as a clear-cell carcinoma derivative but genetically characterized as HGSOC with a TP53 mutation [48]. It has been shown previously that DDR2 promotes metastasis in numerous cancers [16,17,18,19,20,21,22]. High DDR2 expression predicts poor prognosis in ovarian cancer [49]. TWIST1 induces DDR2, which signals through the PI3K/AKT and Src pathways and stabilizes SNAIL1 [25,26,50,51,52]. Interestingly, DDR2 can partially promote metastasis in a kinase-independent manner [40]. However, since we observed an increase in pAKT and pSrc levels in DDR2-depleted POSTN-overexpressing CAFs (Figure 3A), it is likely that the interaction between DDR2 and POSTN is kinase-dependent. Further studies are needed to confirm if DDR2 and POSTN interact in the absence of kinase and/or collagen-binding activity. 

POSTN is a matricellular protein that has been found to affect multiple processes in cancer development [27,28]. Others have shown that POSTN-expressing tumor cells signal through the PI3K/AKT and Src pathways [29,30,31]. We have demonstrated that POSTN-expressing CAFs also can signal through PI3K/AKT and Src (Figure 3A). Tumor-derived POSTN is important for the recruitment of M2-like macrophages during tumor progression [53,54]. In addition to POSTN expression in tumor cells, stromal POSTN has been found to have prognostic significance in various cancers, including ovarian cancer [35,55]. Within the stroma, CAFs are abundant and promote tumor progression, remodel the extracellular matrix, and are responsible for collagen synthesis [56]. In the collagen-abundant stroma, POSTN has been shown to interact with various collagen receptors, including integrins (αv, β1, and β3) and DDR1, however direct binding was not shown [31,32,33,57]. We demonstrated direct binding between POSTN and integrin β1 in CAFs (Figure 5B). Dissecting the functional relevance of this interaction will be important in future work.

In our in vivo study, we show a decrease in tumor burden in mice injected with DDR2-depleted CAFs (CAF shDDR2 + ES2) had decreased tumor burden compared to mice injected with DDR2-expressing CAFs (CAF shSCRM + ES2). In a prior study to decipher the role of DDR2 signaling in CAFs on tumor lung colonization in a breast cancer model, it was observed that CAFs co-injected with tumor cells into mice survived for less than 7 days [40]. Since our in vivo model lasted 15 days, it is possible that CAFs influence tumor initiation and early progression. The kinetics of CAF and tumor cell activity in vivo will be clarified by future studies.

In conclusion, we have identified that DDR2-expressing CAFs regulate POSTN through ITGB1 to promote tumor metastasis in ovarian cancer. DDR2 and POSTN signal through the PI3K/AKT and Src pathway and represent therapeutic targets in ovarian cancer. 

## Supplementary Materials

The following supporting information can be downloaded at: https://www.mdpi.com/article/10.3390/cancers14143482/s1, Figure S1: Tumor spheroid spreading on basement membrane gels is increased in the presence of CAFs. Figure S2A: Characterization of ovarian cancer patient-derived normal omental fibroblast and cancer-associated fibroblasts. Figure S2B: DDR2 depleted CAFs have reduced POSTN levels. We tested this using a second hairpin against DDR2 (shDDR2_2). Figure S2C: Periostin mRNA levels decrease in DDR2-depleted CAFs cultured with tumor cells. Figure S2D: DDR2 does not regulate periostin expression in ovarian cancer cell lines. Figure S3A: DDR2 depletion in CAFs leads to marginal differences in tumor cell attachment in the presence of HPMCs. Figure S3B: Matrigel transwell tumor invasion assay with COV362 cells using conditioned media from DDR2 knockdown and POSTN overexpression CAFs. Figure S4: DDR2’s regulation of periostin increases tumor spreading. Figure S5: Regulatory network including DDR2, periostin and ITGB1. Figure S6: Immunohistochemistry for (A) Stromal POSTN positive and (B) Stromal DDR2 positive cores. 5× images.
