# Supplementary material for: DDR2 Expression in Cancer-Associated Fibroblasts Promotes Ovarian Cancer Tumor Invasion and Metastasis through Periostin-ITGB1
Source: Cancers (Basel). 2022 Jul 18;14(14):3482. doi: 10.3390/cancers14143482 (PMC9319689; doi:10.3390/cancers14143482)
Supplement: Supplementary file 1 [file cancers-14-03482-s001.zip › cancers-1785145-supplementary.pdf]

(A)

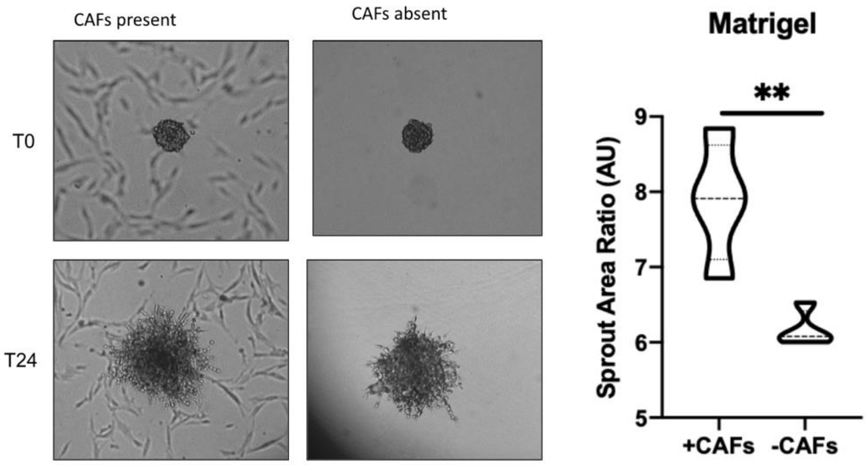
CAFs present
CAFs absent
T0
T24
Matrigel
**
Sprout Area Ratio (AU)
9
8
7
6
5
+CAFs
-CAFs

(B)

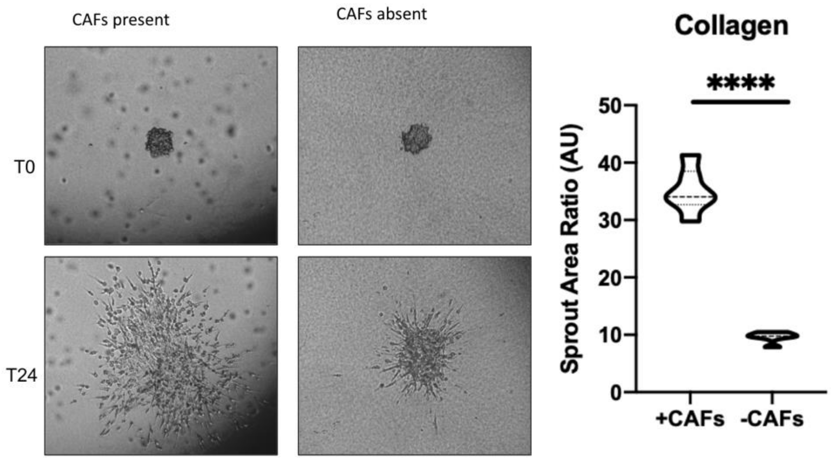
CAFs present
CAFs absent
T0
T24
Collagen
****
Sprout Area Ratio (AU)
50
40
30
20
10
0
+CAFs
-CAFs

(C)

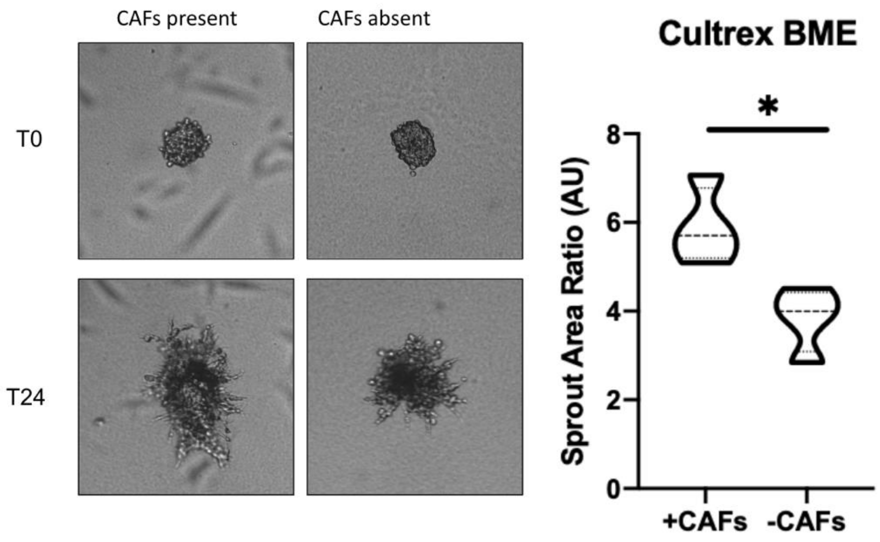

(D)

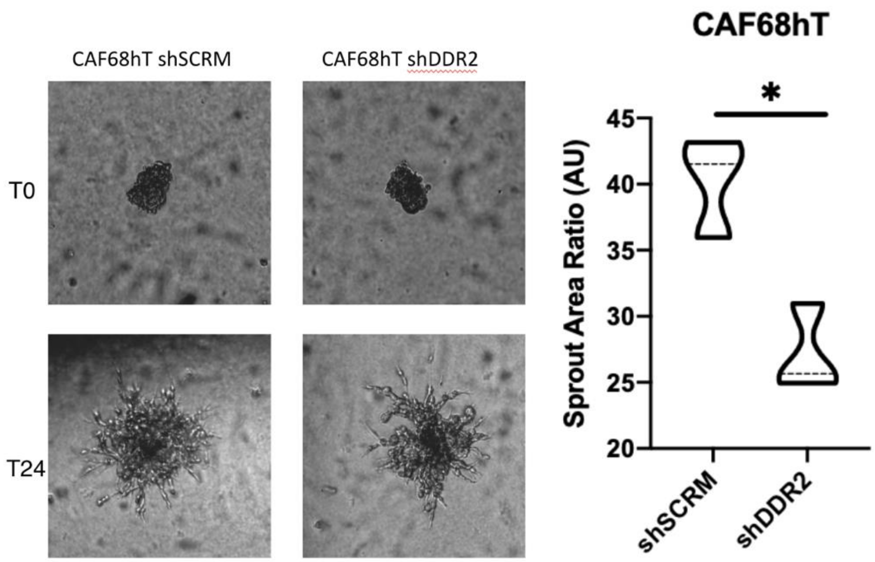

Figure S1. Tumor spheroid spreading on basement membrane gels is increased in the presence of CAFs. This was observed on A) Matrigel, B) Collagen type 1, and C) Cultrex Reduced Growth Factor BME. D) DDR2 expression in CAFs leads to increased tumor spheroid spreading on Matrigel.

(A)

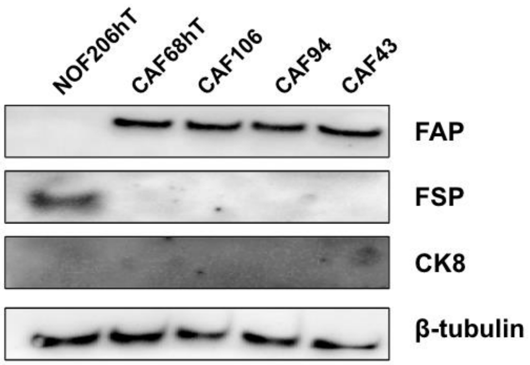
NOF206hT
CAF68hT
CAF106
CAF94
CAF43
FAP
FSP
CK8
β-tubulin

(B)

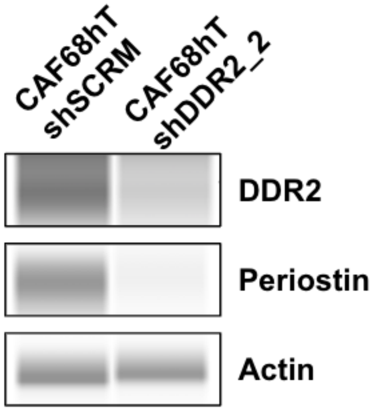
CAF68hT shSCRM
CAF68hT shDDR2_2
DDR2
Periostin
Actin

(C)

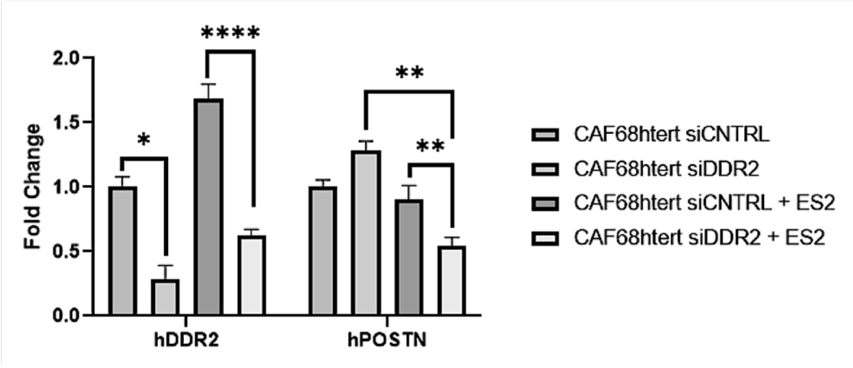

(D)

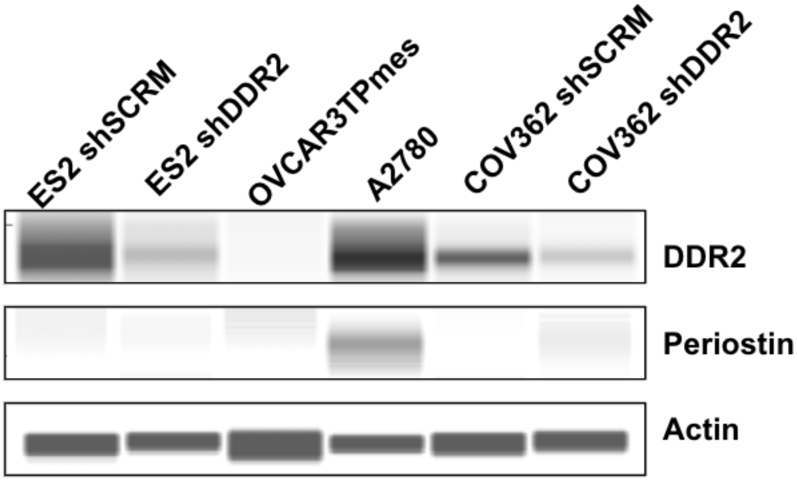

Figure S2. A – Characterization of ovarian cancer patient-derived normal omental fibroblast and cancer-associated fibroblasts, B – DDR2 depleted CAFs have reduced POSTN levels. We tested this using a second hairpin against DDR2 (shDDR2_2), C – Periostin mRNA levels decrease in DDR2-depleted CAFs cultured with tumor cells, D - DDR2 does not regulate periostin expression in ovarian cancer cell lines.

(A)

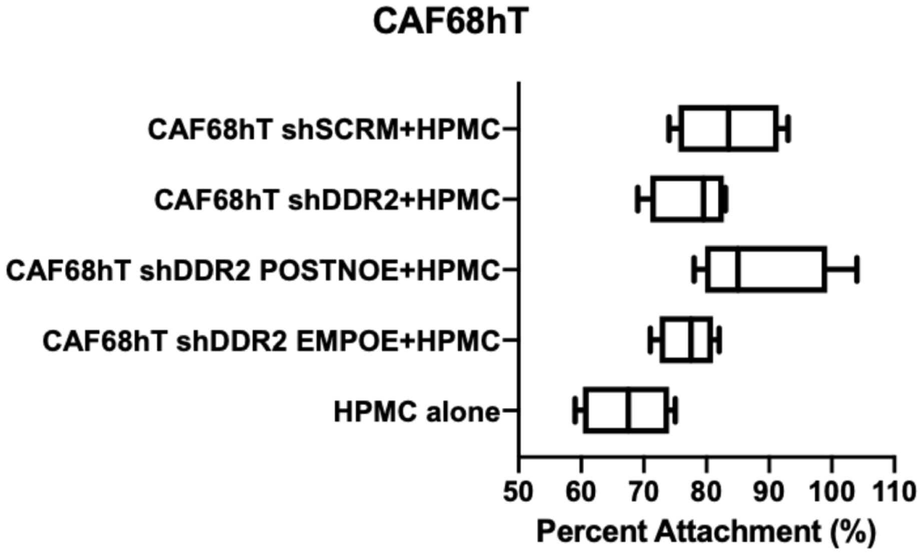

(B)

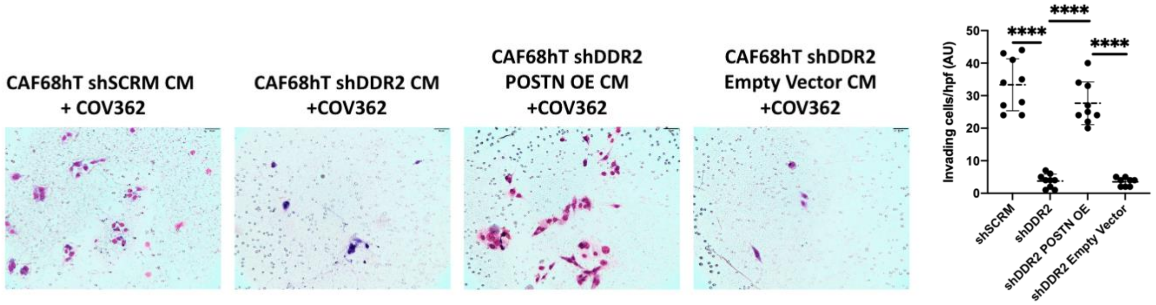

Figure S3. A – DDR2 depletion in CAFs leads to marginal differences in tumor cell attachment in the presence of HPMCs, B – Matrigel transwell tumor invasion assay with COV362 cells using conditioned media from DDR2 knockdown and POSTN overexpression CAFs.

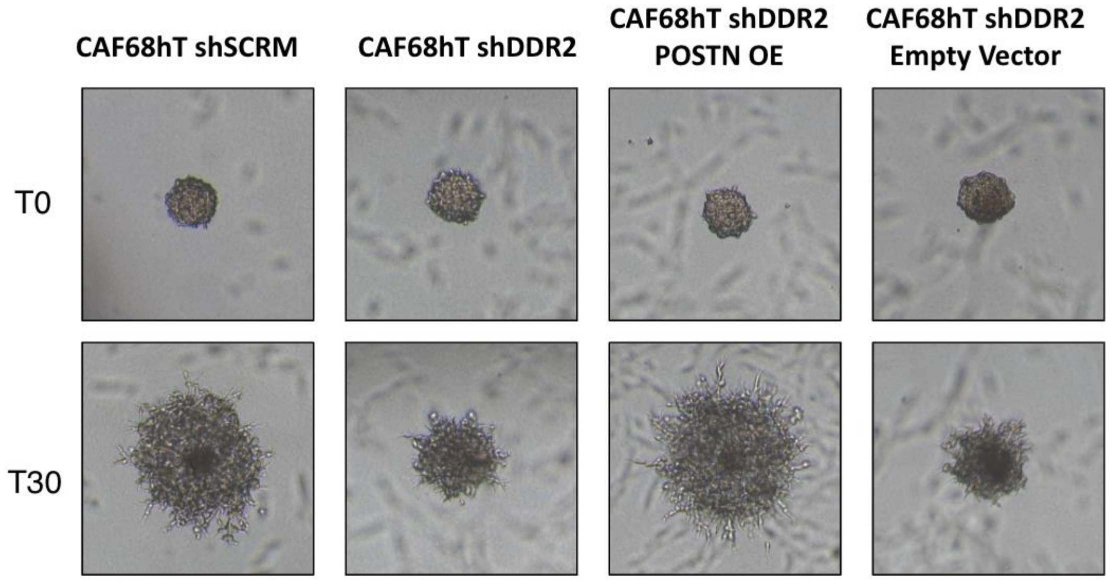

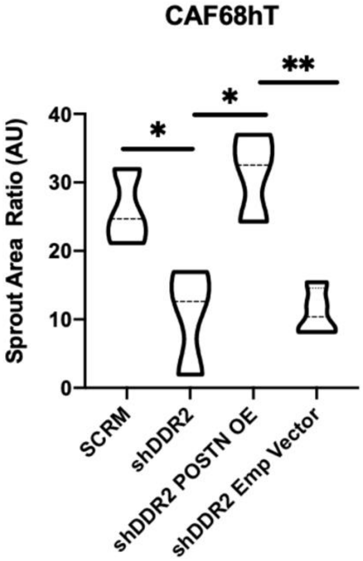

Figure S4. - DDR2's regulation of periostin increases tumor spreading. A) Tumor spreading assay with DDR2-depleted POSTN-overexpressing CAFs in Matrigel, B) Analysis of assay in A.

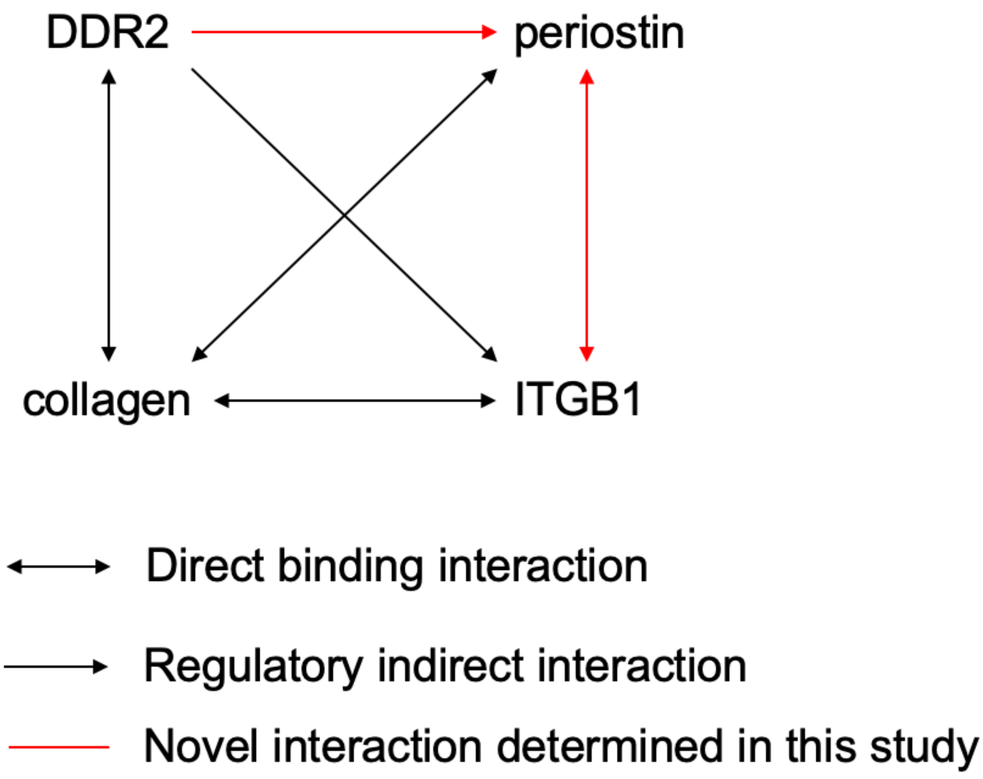

Figure S5. – Regulatory network including DDR2, periostin and ITGB1.

(A)

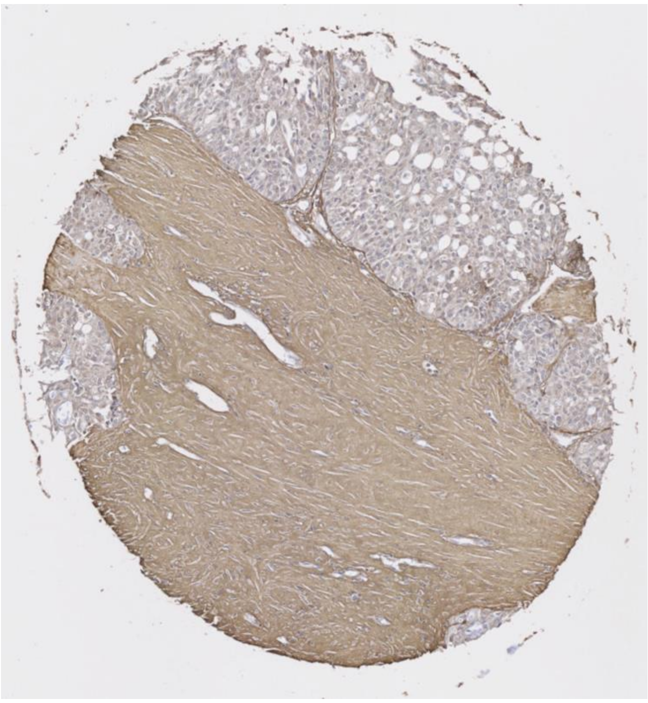

(B)

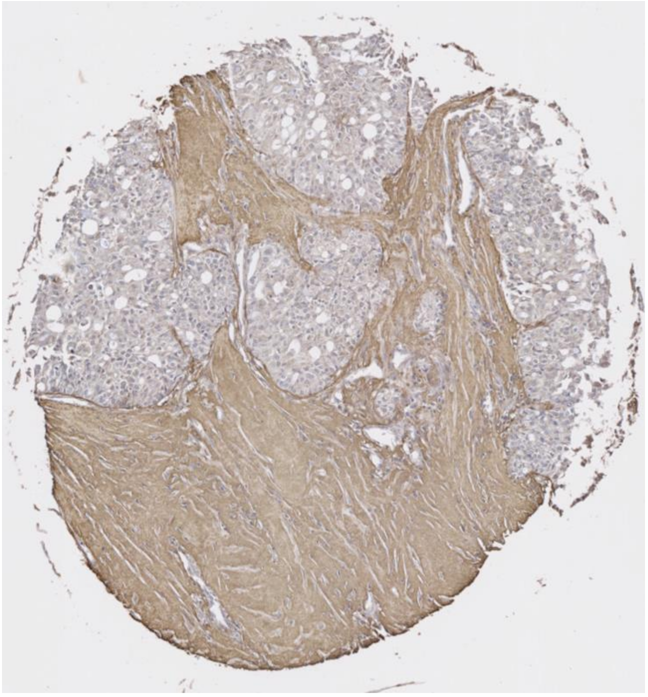

Figure S6. Immunohistochemistry for A) Stromal POSTN positive and B) Stromal DDR2 positive cores. 5X images.
